# Effect the accumulation of bioactive constituents of a medicinal plant (*Salvia Miltiorrhiza* Bge.) by arbuscular mycorrhizal fungi community

**DOI:** 10.1186/s12870-023-04608-x

**Published:** 2023-11-28

**Authors:** Yan-Hong Wu, Yong Qin, Qing-Qing Cai, Min Liu, Dong-Mei He, Xin Chen, Hai Wang, Zhu-Yun Yan

**Affiliations:** 1 State Key Laboratory of Characteristic Chinese Medicine Resources in Southwest China, School of Pharmacy, Chengdu University of Traditional Chinese Medicine, Chengdu, China; 2 School of Medical Technology, Chengdu University of Chinese Medicine, Chengdu, China

**Keywords:** Arbuscular mycorrhizal fungi, *Salvia miltiorrhiza*, Biomass, Tanshinones, Phenolic acids, Biofertilizer

## Abstract

**Background:**

Arbuscular mycorrhizal fungi (AMF) form symbiotic relationships with various terrestrial plants and have attracted considerable interest as biofertilizers for improving the quality and yield of medicinal plants. Despite the widespread distribution of AMFs in *Salvia miltiorrhiza* Bunge's roots, research on the impact of multiple AMFs on biomass and active ingredient accumulations has not been conducted. In this study, the effects of five native AMFs (*Glomus formosanum*, *Septoglomus constrictum*, *Rhizophagus manihotis*, *Acaulospora laevis*, and *Ambispora gerdemannii*) and twenty-six communities on the root biomass and active ingredient concentrations of *S. miltiorrhiza* were assessed using the total factor design method.

**Results:**

Thirty-one treatment groups formed symbiotic relationships with *S. miltiorrhiza* based on the pot culture results, and the colonization rate ranged from 54.83% to 89.97%. AMF communities had higher colonization rates and total phenolic acid concentration than single AMF, and communities also appeared to have higher root fresh weight, dry weight, and total phenolic acid concentration than single inoculations. As AMF richness increased, there was a rising trend in root biomass and total tanshinone accumulations (ATTS), while total phenolic acid accumulations (ATP) showed a decreasing trend. This suggests that plant productivity was influenced by the AMF richness, with higher inoculation benefits observed when the communities contained three or four AMFs. Additionally, the affinities of AMF members were also connected to plant productivity. The inoculation effect of closely related AMFs within the same family, such as *G. formosanum*, *S. constrictum*, and *R. manihotis*, consistently yielded lower than that of mono-inoculation when any combinations were applied. The co-inoculation of *S. miltiorrhiza* with nearby or distant AMFs from two families, such as *G. formosanum*, *R. manihotis*, and *Ac. laevis* or *Am. gerdemannii* resulted in an increase of ATP and ATTS by more than 50%. AMF communities appear to be more beneficial to the yield of bioactive constituents than the single AMF, but overall community inoculation effects are related to the composition of AMFs and the relationship between members.

**Conclusion:**

This study reveals that the AMF community has great potential to improve the productivity and the accumulation of bioactive constituents in *S. miltiorrhiza*, indicating that it is an effective way to achieve sustainable agricultural development through using the AMF community.

**Supplementary Information:**

The online version contains supplementary material available at 10.1186/s12870-023-04608-x.

## Introduction

Soil microbes perform numerous crucial ecological services for both natural and agricultural ecosystems. It is widely recognized that the inoculation of beneficial soil microbial communities is a vital way to improve plant productivity and the sustainability of agricultural ecosystems [1]. Arbuscular mycorrhizal fungi (AMF), a group of soil-dwelling fungi, formed a symbiotic relationship with approximately 80% of terrestrial plants for thousands of years [2]. This symbiotic relationship facilitated access to nutrients that were not readily available [3, 4], improved water uptake [5], and increased plant resistance to adversity such as drought, salinity, and pathogens [6–8]. Additionally, the relationship between AMF and plants often improved crop yield [9, 10] and the accumulation of secondary metabolites and bioactive components in medicinal and aromatic plants [11, 12]. However, the influence of AMF on medicinal plants' growth and secondary metabolites is contingent upon plant genotypes, AMF strains, and environmental conditions [13]. For instance, when inoculated with *Glomus mosseae*, *G. intraradices*, *G. versiform*, *G. geosporum,* and other five Glomus species, *Calendula officinalis -*L. showed different growth, physiology, phytochemistry, and nutrient absorption characteristics [14]. Therefore, it is essential to investigate the effects of various AMF inoculations on the growth and metabolism of aromatic plants.

*Salvia miltiorrhiza* Bunge (Lamiaceae, Salvia), also known as Danshen, has demonstrated notable antioxidant, antibacterial, anticoagulant, and anti-tumor activities and has been commonly used to treat cardiovascular diseases for almost 2,000 years [15–17]. As a mycorrhizal-dependent medicinal plant, *S. miltiorrhiza* can form a good symbiosis with various AMFs [18–20]. The inoculation of either *Acaulospora bireticulata* or *G. versiforme* increases the content of salvianolic acid B (SB), tanshinone IIA (TS-IIA), and dihydrotanshinone I (DT-I) in *S. miltiorrhiza* root [18, 19]. In addition, various AMFs of the Glomus genus significantly promoted the growth and the accumulation of N, P, K, and active ingredients of *S. miltiorrhiza*, with inoculation with *G. versiforme* exhibiting the most significant effect [20]. Despite the great potential of AMF in the growth-promoting effect, most current studies are still limited to the beneficial effects of single AMF on growth and active ingredient accumulation, and there is a lack of systematic studies on inoculating AMF communities. The functional diversity among different strains of diverse and rich microbial communities suggests their tremendous research potential compared to mono-inoculums, enabling optimal utilization of limited resources [21, 22]. Since both the structure and diversity of microbial communities significantly affect the productivity of plants [23], it is necessary to explore the impact of varied AMF combinations on the growth and metabolism of *S. miltiorrhiza*.

Five native AMFs, *G. formosanum*, *Septoglomus constrictum*, *Rhizophagus manihotis*, *Ac. laevis*, and *Ambispora gerdemannii*, were chosen for pot experimentation in greenhouse to investigate individual AMFs and combined species on the root biomass and the accumulation of nine active ingredients, including danshensu sodium (DS), caffeic acid (CA), rosmarinic acid (RA), SB, salvianolic acid A (SA), DT-I, cryptotanshinone (CT), tanshinone I (TS-I), and TS-IIA. The following objectives were achieved through the greenhouse experiment: 1) to study the effects of different native AMF and their different combinations of communities on the root growth and active ingredients of *S. miltiorrhiza*, 2) to explore the correlation between AMF species richness and affinity with the formation of the economic yield and determine the key of AMF as biofertilizer in the growth and metabolism, and 3) to identify the combinations of communities that are favorable to the yield of the medicinal parts and the accumulation of active ingredients of *S. miltiorrhiza*. This study shows that the composite mycorrhizal technology, as a means of biotechnology, promotes growth and increases bioactive compound yields in *S. miltiorrhiza*.

## Materials and methods

### Preparation of plant materials and AMF inoculum

**Fig. 1:**
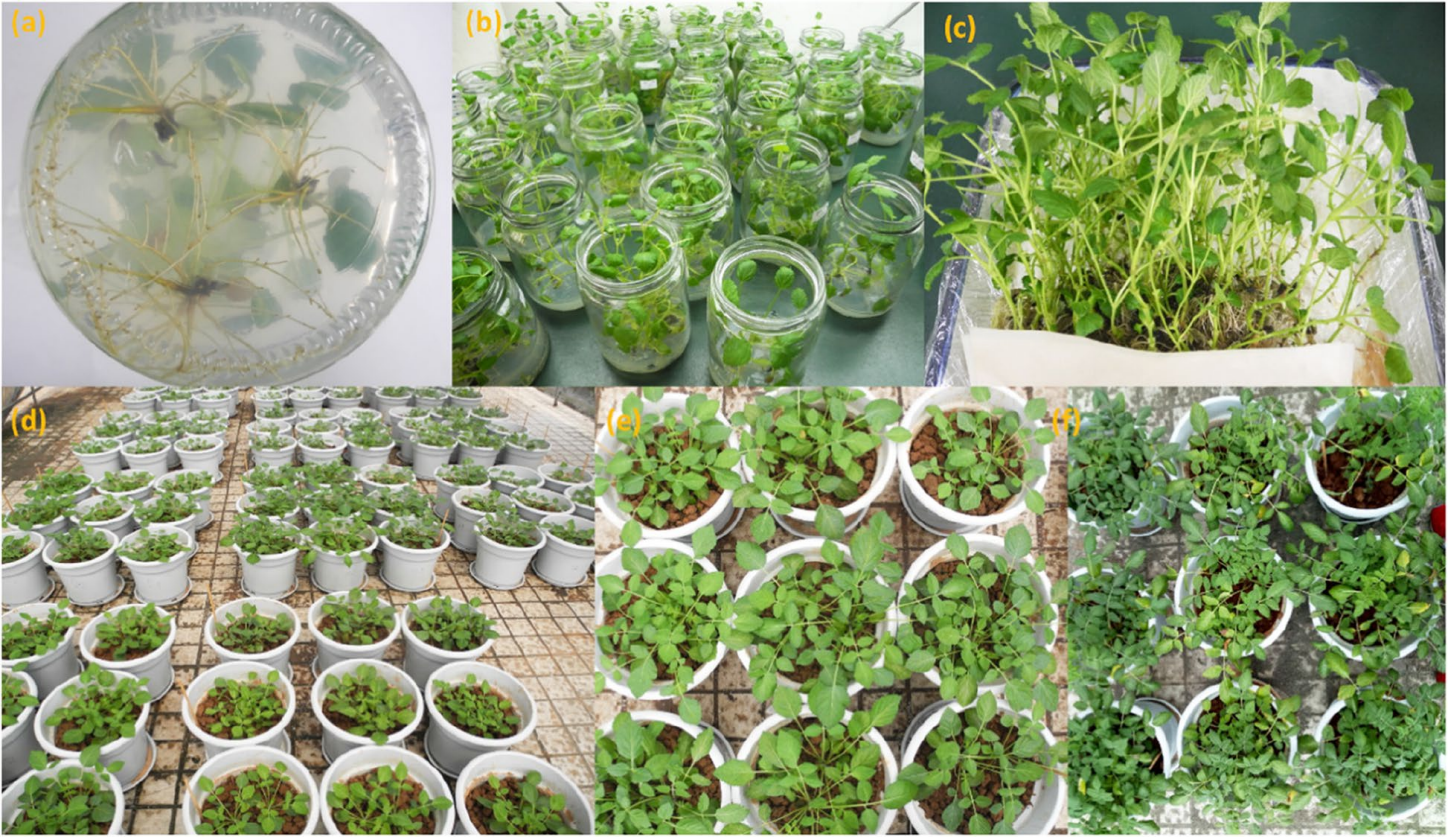
The growth of *S. miltiorrhiza* at different stages. (**a**) Root development of transplantable tissue culture seedlings; (**b**) The tissue culture seedlings are exposed to the air to adapt to the environment; (**c**) Part of the tissue culture seedlings would be transplanted; (**d**) Placement of some potted plants in the greenhouse; (**e**) Growth of *S. miltiorrhiza* in ten weeks after transplanting; (**f**) Growth of *S. miltiorrhiza* in twenty-two weeks after transplantation

The plant materials were obtained from the Medicinal Botanical Garden of Chengdu University of Traditional Chinese Medicine, and the plant was identified as *S. miltiorrhiza* Bge. cv. Sativa by Professor Xin Chen (Chengdu University of Traditional Chinese Medicine, College of Pharmacy). The plant sample is housed at the State Key Laboratory of Characteristic Chinese Medicine Resources in Southwest China, with a number of SMDY17. The tender leaves of *S. miltiorrhiza* were cultivated through tissue culture procedures to produce genetically homogeneous seedlings in test tubes, with the aim of eliminating irrelevant influence of endophytes in plant seeds [24]. The medium for test-tube seedlings was prepared as follows: callus induction medium was MS + 2.0 mg∙L^−1^ 6-BA + 1.0 mg∙L^−1^ NAA + 30 g sucrose; differentiation medium was MS + 1.0 mg∙L^−1^ 6-BA + 0.1 mg∙L^−1^ NAA + 30 g sucrose; rooting medium was 1/2 MS + 0.2 mg∙L^−1^ NAA + 0.5 mg∙L^−1^ IBA + 15 g sucrose. A rooting rate of 94% was achieved. Plants with 5 cm roots were used as the material of experimental pot plants (Fig. 1a).

In our previous research, five AMFs were isolated from *S. miltiorrhiza* planting base of eight provinces in China by Liu et al. [25]. Five AMFs, *Glomus formosanum* Wu & Chen (hereafter referred to as A), *Septoglomus constrictum* (Trappe) Sieverd., Silva & Oehl (hereafter referred to as B), *Rhizophagus manihotis* (Sieverd. & Schenck) Walker & Schuessler (hereafter referred to as C), *Acaulospora laevis* Gerd. & Trappe (hereafter referred to as D), *Ambispora gerdemannii* (Rose et Daniels & Trappe) Walker et Vestberg & Schuessler (hereafter referred to as E), which including one class, three orders, three families, and five genera, were used in this study. The AMFs were stored at the State Key Laboratory of Characteristic Chinese Medicine Resources in Southwest China, Chengdu, China, with a number of DSAM01, DSAM03, DSAM05, DSAM06, and DSAM07.

The five AMFs were propagated individually on *Trifolium repens* L. using autoclaved fine sand as a substrate in the greenhouse. After two months, the plants were harvested, and wet sieving, decanting, and stereomicroscopy were used to measure the number of spores [26]. The cultured sand acts as an inoculum which contains infected root fragments, hyphae, and spores.

The substrate used in the pot experiment consisted of autoclaved soil, which was collected from the experimental field "the base of Danshen in Meishan village" in Zhongjiang County (Sichuan, China: 30^◦^57′ 6'' N, 104^◦^33′ 17'' E). Before the test, composite soil samples were collected at a depth of 0 ~ 30 cm to determine their physical and chemical characteristics: alkali-hydrolysable N 128.93 mg∙kg^−1^, available K 43.50 mg∙kg^−1^, available P 34.71 mg∙kg^−1^, and organic matter 35.1 mg∙kg^−1^, and pH (H_2_O) 7.46.

### Experimental design

**Fig. 2:**
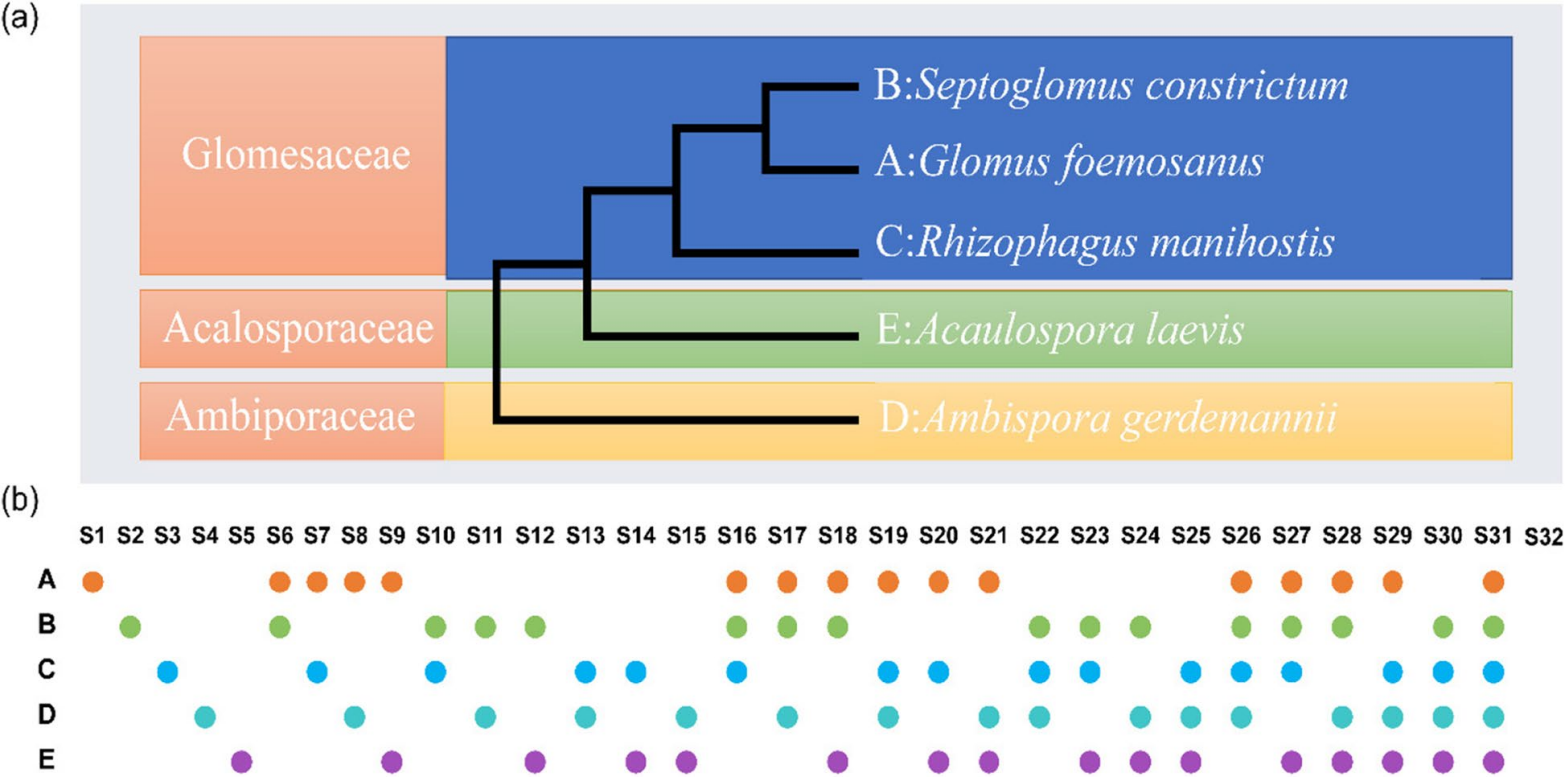
Phylogenetic distance and grouping settings of five AMFs. (**a**) Phylogenetic diagram of the five candidate AMFs; (**b**) Distribution of the five AMFs in each treatment group. A, B, C, D, and E represent *Glomus formosanum*, *Septoglomus constrictum*, *Rhizophagus manihotis*, *Acaulospora laevis*, *Ambispora gerdemannii*, respectively, and the distribution in different samples was represented by orange, green, blue, blue-green and purple circles, respectively. S1-S32 represents different inoculation treatments

The experiments were conducted in the greenhouse of the Chengdu University of Traditional Chinese Medicine greenhouse, Chengdu, China. The experiment was conducted in pots filled with 5 kg of sterilized substrate. The sieved substrate (pore size, 5 mm) was hermetically sterilized with Dazomet 250 g∙M^3^ for one week to kill the soil microorganisms. A sterility test was negative before use. According to the classification method of Redecker et al. [27], the phylogenetic relationship diagram of five AMF species is shown in Fig. 2a. To study the inoculation effects of different AMFs and various possible combinations on the growth of *S. miltiorrhiza*, we designed thirty-one different inoculation treatments using the method of complete factorial design. The inoculums of different treatments were evenly mixed in equal amounts to prepare 200 spores per 10 g of soil according to the design scheme in Fig. 2b.

Pots (20 cm in diameter and 30 cm in height) were autoclaved (121℃ for 30 min) and filled with 3 kg of sterilized substrate. After being exposed to the environment for one week of adaptation, the plants were transplanted for pot experiments (Fig. 1b and c). For each treatment pot, 20 g of inoculum was distributed around the roots of test tube seedlings (two plants per pot) and then covered with a further 2 kg of sterilized soil. One group was not inoculated with inoculums as a non-mycorrhizal control (hereafter referred to as CK). Sterile water was poured over the plants. Pots were randomized entirely in the glasshouse and grown at the natural temperature and moisture for the rest of the culture period until harvest. All treatments were watered with 1/2 MS nutrient solution (without agar and sugar) once every two weeks during the greenhouse period. The growth of *S. miltiorrhiza* plants in pots of greenhouse is shown in Fig. 1d, e, and f.

### Plant harvesting and determination of colonization rates

Three replicate pots for each treatment were harvested after a growth period of twenty-four weeks, and part of the root growth is shown in Fig. S1. The roots were washed from the substrate with deionized water and divided into three parts. One part was dried at 105℃ for 48 h and weighed; one part was immersed in liquid nitrogen, stored at -80℃ for analyzing the active substance concentrations; and one part, primarily consisting of the root tip, was used for the determination of mycorrhizal colonization.

According to the method based on Phillips & Hayman (1970) [28] and Brundrett et al. [29], the colonization intensity of mycorrhiza was determined. Briefly, roots were washed with tap water, digested with 8% KOH in a water bath at 90℃ for 40 min, rinsed three times with water, acidified with 2% HCl at room temperature for 5 min, and stained with 0.01% fuchsin lactic acid in a water bath at 90℃ for 40 min. Mycorrhizal colonization was estimated under an optical microscope (OLYMPUS CH20 BIMF200, Japan), considering the presence or absence of fungal structures in the 1 cm long root segments. The percentage of the mycorrhizal root was calculated by using the grid-line intersection method [30], and the mycorrhizal dependency (MD) was determined using the method of Liu et al. [31].

### Extraction and analysis of the active ingredients

**Fig. 3:**
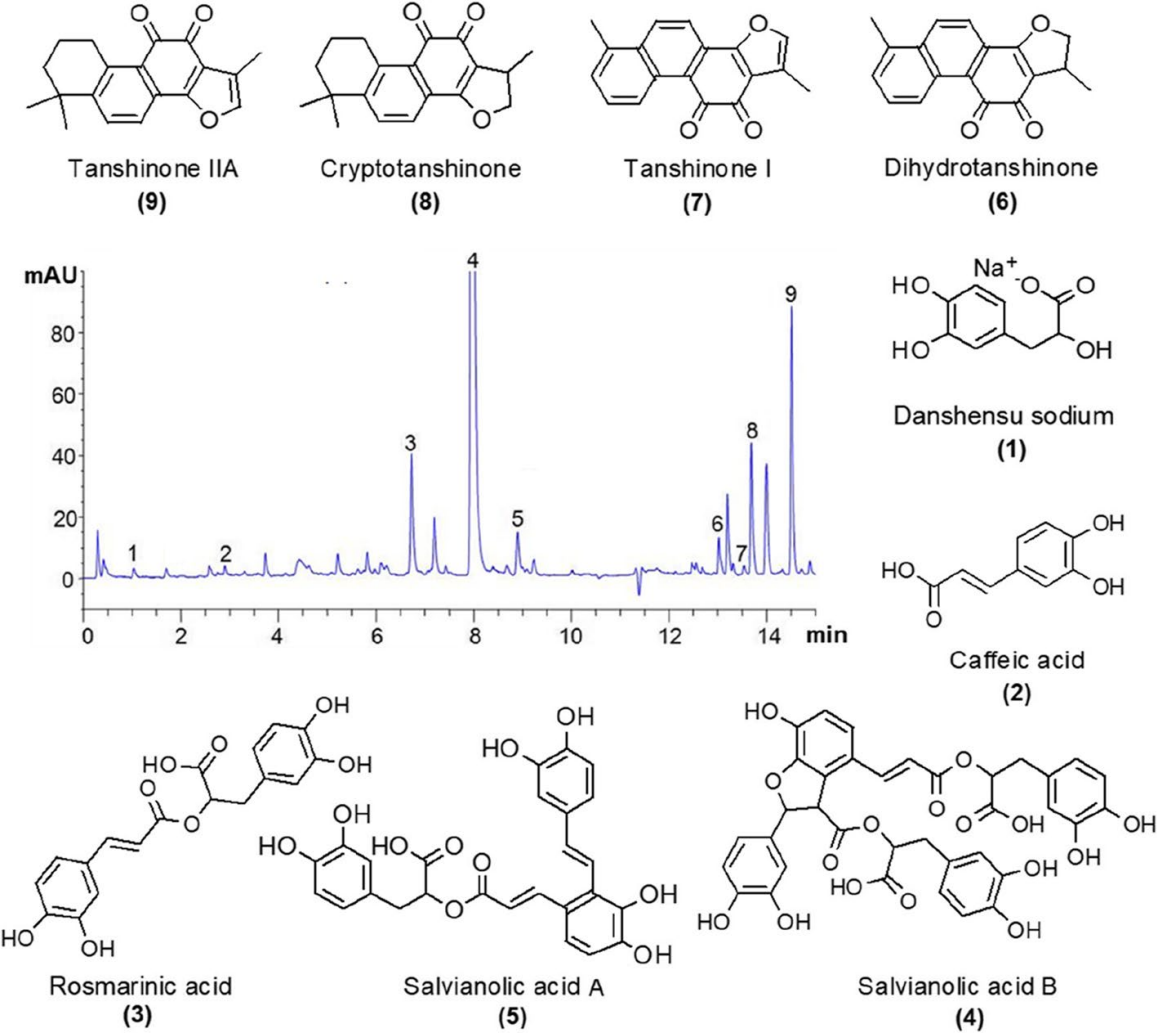
Nine secondary metabolites induced in *S. miltiorrhiza*. (1) Danshensu sodium, DS; (2) Caffeic acid, CA; (3) Rosmarinic acid, RA; (4) Salvianolic acid B, SB; (5) Salvianolic acid A, SA; (6) Dihydrotanshinone I, DT-I; (7) Tanshinone I, TS-I; (8) Cryptotanshinone, CT; (9) Tanshinone IIA, TS-IIA

For sample preparation, a modified extraction procedure is used in this work to analyze native phytochemicals and avoid any drying treatment to exclude the changes of secondary metabolites present in the homogenous samples. Nine active compounds, DS, CA, RA, SA, SB, DT-I, CT, TS-I, and TS-IIA, were measured in this study (Fig. 3). The frozen roots were crushed with liquid nitrogen and extracted with 90% methanol. Approximately 500 mg of root powder was dissolved in 90% methanol and ultrasonically extracted at 40℃ for 45 min, with the weight of the extraction solution consisting of methanol. After centrifugation (1000 rpm, 10 min, 25℃), the supernatant was collected and filtered through a 0.22 *μ*m diameter microporous membrane for analysis. UPLC-DAD analyses were performed on an Agilent UPLC system (Agilent 1290, Agilent Corp). Analyses were performed on a 2.1 × 50 mm, 1.8 µm Eclipse Plus C18 column (ZORBAX, Agilent, USA) with a column temperature of 35℃and a sample manager temperature of 10℃. For the determination of nine compounds, each sample (2 *μ*L) was analyzed by eluting with a mixture of 0.02% (v/v) phosphoric acid in water (solvent A) and acetonitrile (solvent B) in a linear gradient with the following conditions (flow rate: 1 mL·min^−1^), which was modified based on the method of Li et al. [32]. The gradient condition was as follows: initial conditions 95% A; from 0 to 0.5 min, 95% A; from 0.5 to 2 min, from 95 to 87% A; from 2 to 6.5 min, from 87 to 78% A; from 6.5 to 10.0 min, from 78 to 72% A; from 10.0 to 11.5 min, from 72 to 40% A; from 11.5 to 15.0 min, from 40 to 10% A; from 15.0 to 20.0 min, back to 95% A, followed by a period of 5 min for column equilibration period. Spectral data from the DAD detector were analyzed during the whole run at 265 nm, 280 nm, and 330 nm. Each constituent was identified by comparing the peaks obtained in the different chromatograms with the retention time and UV spectra of standards under the same chromatographic conditions.

The amount of the marker compounds was determined by the standard external method using five concentration levels for each compound. Standard calibration curves were plotted over a range of concentrations. An aliquot of each standard compound was analyzed in triplicate under the same conditions at extracts using the Agilent UPLC-DAD system. The standard solutions of the authentic samples were dissolved in methanol. The retention times, UV absorbance, linear regression equations, and correlation coefficients of the external standards are shown in Table 1. The above analyte was quantified by the standard calibration curve method.

**Table 1:** The calibration curve data of reference substance in *S. miltiorrhiza* root

	Linear range (ng)	Regression equation	*R*^2^
DS	0.560 ∼ 8.960	y = 11.32557x-0.98662	0.99991
CA	0.168 ∼ 4.480	y = 56.20537x-6.85365	0.99996
RA	3.420 ∼ 68.400	y = 20.96961x-22.24801	0.99985
SB	91.60 ∼ 732.80	y = 6.62872x-44.93137	0.99997
SA	0.384 ∼ 10.240	y = 22.17195x-6.96602	0.99992
DT-I	0.204 ∼ 10.880	y = 58.09684x-10.34958	0.99984
TS-I	0.020 ∼ 0.672/	y = 72.97334x + 1.66830	0.99990
CT	1.470 ∼ 78.400	y = 38.68225x-27.81167	0.99999
TS-IIA	0.730 ∼ 24.400	y = 63.73954x-29.18675	0.99997

### Data analysis

The percentage of root length colonized by AMF hyphae, arbuscules, and vesicles were expressed as the ratio of sections with these structures to all root sections per hundred sample. One-way analysis of variance (ANOVA) was performed on plant biochemical data between treatments after assessing the homogeneity of variances with the Levene test, where necessary data were transformed to homogenize variance. Significant differences between treatments were confirmed using multiple comparisons by the LSD method at the 5% significance level. When variances were unequal, the Games-Howell pair-wise comparison test or the Moses test of extreme reactions was used. Statistical analysis and general linear model analysis were performed using SPSS 21.0 software (IBM, Armon, NY, USA). Correlations between the traits were calculated using Pearson’s coefficient using R Studio through the ‘corrplot’ package (https://www.r-project.org).

## Results

### Mycorrhizal colonization

**Fig. 4:**
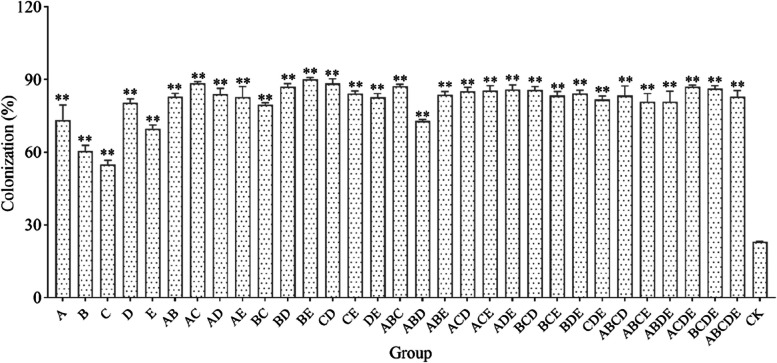
The colonization of different groups in *S. miltiorrhiza* root compared with CK. "**" indicates a highly significant difference (*p* < 0.01) when compared with CK. Different compositions of thirty-two groups were used in this study as Fig. 2b

**Fig. 5:**
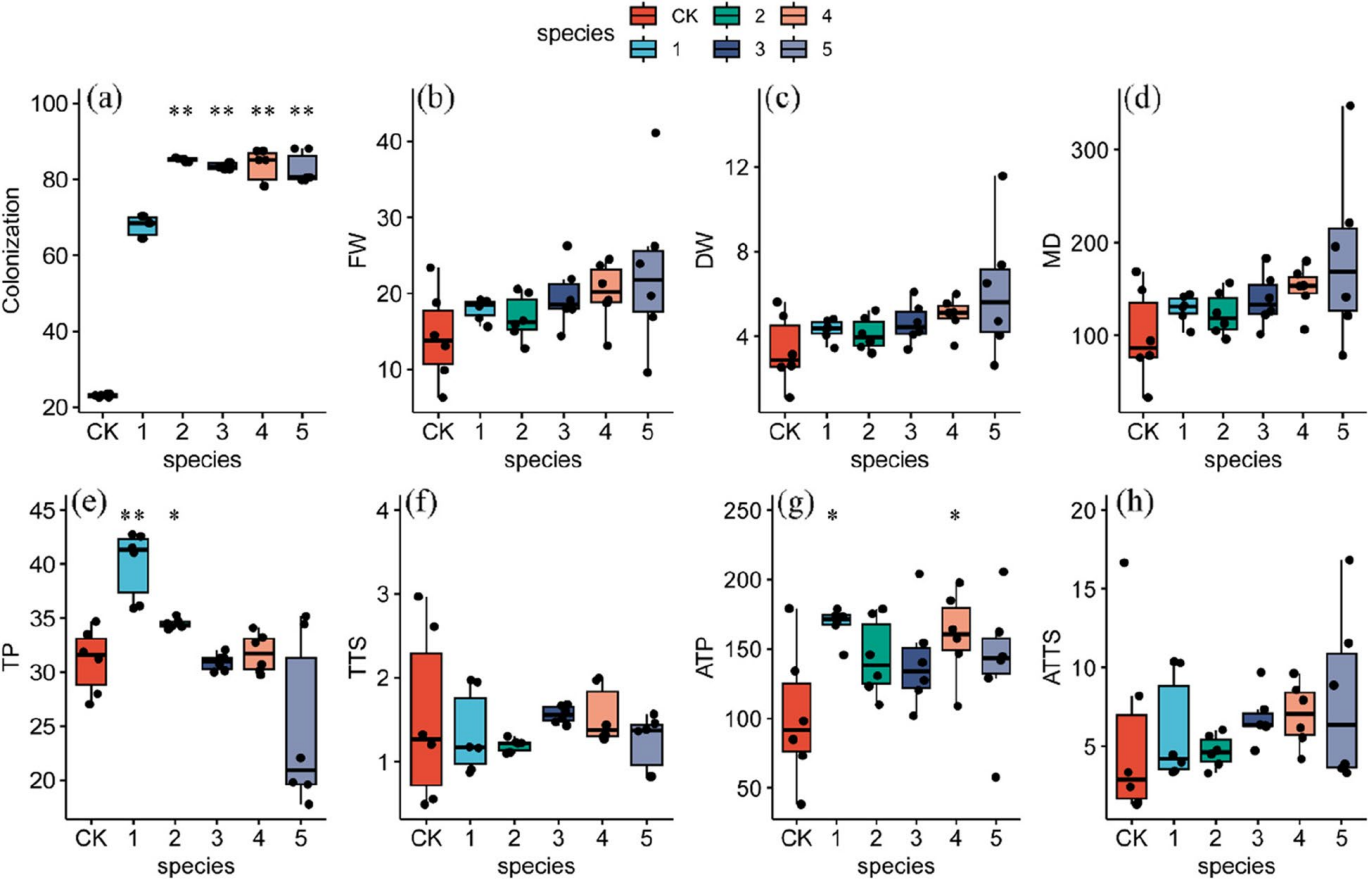
Relationship among AMF species richness and colonization (**a**), FW (**b**), DW (**c**), MD (**d**), TP (**e**), TTS (**f**), ATP (**g**), and ATTS (**h**). The number of species represents the number of AMFs contained in the inoculum. "*" indicates a significant difference (*p* < 0.05), and "**" indicates a highly significant difference (*p* < 0.01) when compared with CK

All inoculation treatments successfully colonized the roots of *S. miltiorrhiza* (Fig. 4). The percentage of root colonization varied between the inoculation treatments, ranging from 54.8% to 90.0%. With the increase in AMF species richness, it was easy to see that the community possessed had a higher colonization rate than a single AMF when there were more than two AMF species in the community (Fig. 5a). However, the treatment based on sterilization substrate (CK) showed a colonization rate of 23.0%, which might point to fungal contamination from airborne flotsam during the pot experiments.

### Plant biomass

**Fig. 6:**
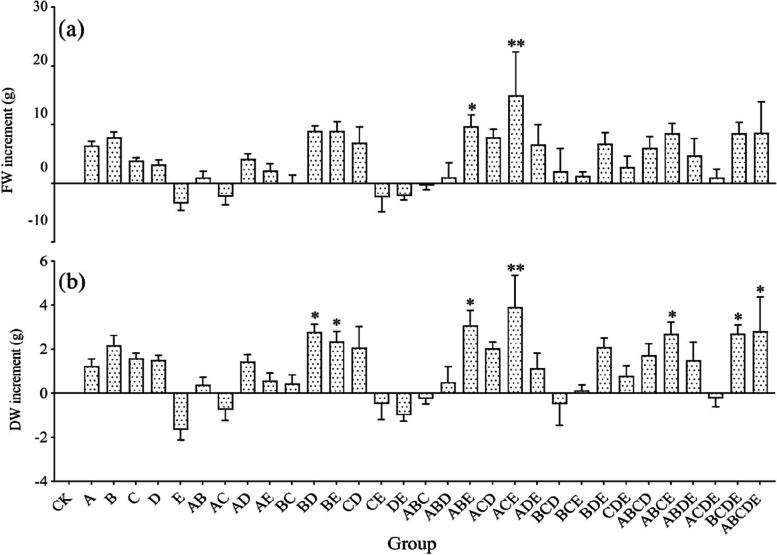
The increase of FW and DW in *S. miltiorrhiza* root compared with CK. (**a**) plant root fresh weight (FW), and (**b**) plant root dry weight (DW). Different compositions of thirty-two groups were used in this study as Fig. 2b. "*" indicates a significant difference (*p* < 0.05), and "**" indicates a highly significant difference (*p* < 0.01) when compared with CK

AMF inoculums had a promotion effect on most roots' biomass of *S. miltiorrhiza* (Table 2). However, the level to which plant growth was enhanced or decreased varied between the inocula (Fig. 6). A mono-inoculation of strain E reduced the roots' fresh weight (FW) and dry weight (DW). In contrast, inoculation with strains A, B, C, or D increased the FW and DW, of which B had the most potent enhancing effect, with increases of 54.8% and 65.1%, respectively. Interestingly, the DW of the communities AB, AC, and BC (composed of the same family) was lower than that of the mono-inoculation treatments (strains A, B, or C). When the two AMFs in the community were from different families, the FW and DW in the roots show a combination difference. For instance, the root biomass of *S. miltiorrhiza* increased when the inoculation of strain E was combined with strains A or B but decreased when combined with strains C or D.

**Table 2:** Effect of AMF inoculation on morphological characteristics of *S. miltiorrhiza* root

Group	FW (g)	DW (g)	MD (%)
A	20.77 ± 5.64	4.56 ± 1.14	143.38
B	22.18 ± 5.46	5.50 ± 1.95	165.60
C	18.22 ± 5.64	4.92 ± 1.59	150.30
D	17.52 ± 6.33	4.83 ± 1.55	151.44
E	10.88 ± 4.03	1.68 ± 0.50	54.42
AB	15.32 ± 5.73	3.71 ± 1.69	116.16
AC	12.07 ± 2.80	2.58 ± 0.58	83.07
AD	18.48 ± 7.98	4.76 ± 2.15	146.54
AE	16.48 ± 7.14	3.90 ± 2.18	113.83
BC	14.33 ± 5.48	3.76 ± 1.50	115.38
BD	23.22 ± 7.84	6.11 ± 2.01^*^	197.29
BE	23.20 ± 8.93	5.68 ± 2.29^*^	175.07
CD	21.25 ± 9.46	5.40 ± 2.71	165.37
CE	11.98 ± 6.25	2.85 ± 1.86	85.90
DE	12.18 ± 6.77	2.34 ± 1.16	78.33
ABC	14.02 ± 5.16	3.08 ± 1.22	96.48
ABD	15.37 ± 8.58	3.82 ± 2.42	121.29
ABE	24.03 ± 6.08^*^	6.41 ± 1.97^*^	200.94
ACD	22.18 ± 8.31	5.37 ± 2.00	165.66
ACE	29.30 ± 22.85^**^	7.24 ± 4.72^**^	241.09
ADE	20.93 ± 9.45	4.45 ± 2.55	146.31
BCD	16.37 ± 11.45	2.84 ± 1.89	103.80
BCE	15.60 ± 4.92	3.46 ± 1.56	104.39
BDE	21.10 ± 10.48	5.42 ± 2.58	168.65
CDE	17.12 ± 9.87	4.12 ± 2.35	131.60
ABCD	20.37 ± 9.54	5.06 ± 2.32	160.59
ABCE	22.85 ± 5.43	6.03 ± 1.52^*^	184.82
ABDE	19.03 ± 4.61	4.82 ± 1.24	149.31
ACDE	15.32 ± 6.52	3.11 ± 1.07	98.12
BCDE	22.87 ± 6.19	6.04 ± 1.66^*^	187.27
ABCDE	22.90 ± 10.64	6.14 ± 3.16^*^	185.93
CK	14.33 ± 6.13	3.33 ± 1.68	100.00

*FW* Fresh weight, *DW* Dry weight, *MD* Mycorrhizal dependency. Different compositions of thirty-two groups were used in this study as Fig. 2b. "*" indicates a significant difference (*p* < 0.05), and "**" indicates a highly significant difference (*p* < 0.01) when compared with CK

In addition, the root FW and DW of three AMFs composed of the same family (community ABC) decreased by 21.7% and 7.5% compared to CK, respectively. On the contrary, the mixed inocula of three families (such as communities ADE, BDE, and CDE) increased the biomass. The FW and DW increased in close family combinations (e.g., communities ABE, ACE, and BCE) and the most distant family combinations (e.g., communities ABD and ACD). When *S. miltiorrhiza* was co-inoculated with more than three AMFs, the effect of ABCDE strongly promoted the FW and DW (59.8% and 84.4% higher than CK, respectively), and both communities ABCE and BCDE significantly increased DW by more than 80% compared to the CK (*p* < 0.05). Notably, when the affinities of AMF composition were too dispersed (e.g., community ACDE), plant root FW and DW showed a decrease.

In addition, the FW and DW were influenced by whether AMF was inoculated and the richness of the inoculated community species (Fig. 5b and c). With the increase of AMF species, there was an increasing trend in the FW and DW; the maximum average DW accumulation was reached at the community species richness of five species, which was about 59.8% higher than that of CK. Interestingly, the mono-inoculum seemed to be more advantageous than the double inoculation and possessed higher FW and DW. As an important indicator, MD is often used to measure the symbiotic effect of mycorrhizal inoculation and growth promotion. The results showed that the MD of fourteen groups reached more than 150% (Table 2), and the community ACE had the most significant promoting effect (MD = 241.09%). With the increase of AMF species, the MD of plants showed an increasing trend (Fig. 5d). It seems that *S. miltiorrhiza* formed an excellent symbiotic relationship with AMF(s) and promoted the accumulation of root biomass (especially community ACE). Similarly, the inoculation effects of different AMF(s) varied, but most showed growth-promoting effects. The inoculation effects of AMF communities were related to the AMF species richness in the community and their genetic relationship.

### The concentration of the active substances

**Fig. 7:**
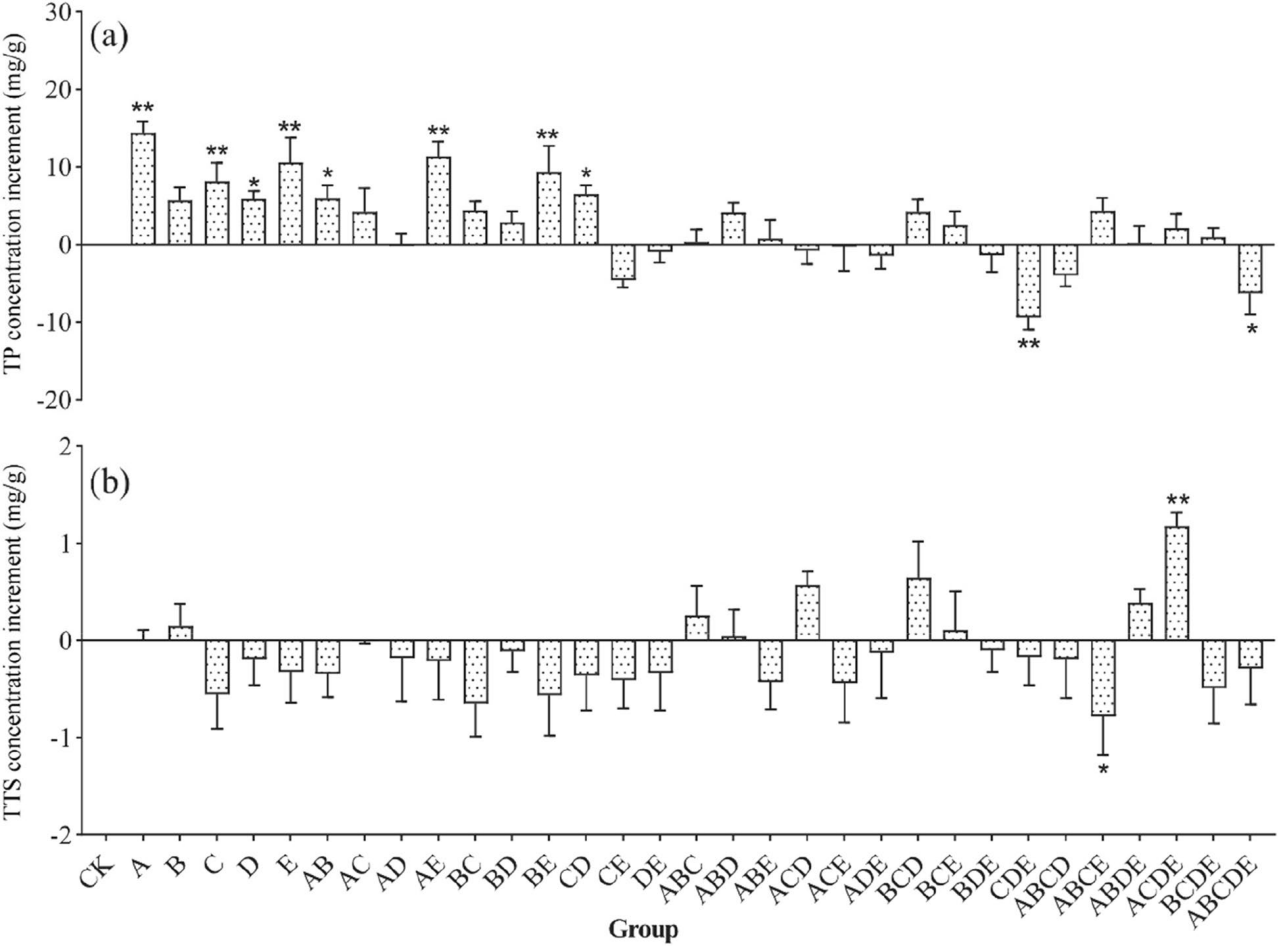
The increase of TP and TTS concentrations in *S. miltiorrhiza* roots compared with CK. (**a**) the concentration of TP, and (**b**) the concentration of TTS. Different compositions of thirty-two groups were used in this study as Fig. 2b. "*" indicates a significant difference (*p* < 0.05), and "**" indicates a highly significant difference (*p* < 0.01) when compared with CK

Hydrophilic and lipophilic components are a series of bioactive substances in *S. miltiorrhiza* root with different pharmacological activities. Nine active components, including DS, CA, RA, SB, SA, DH-I, TS-I, TS-IIA, and CT, were detected simultaneously by UPLC, and typical chromatograms are shown in Fig. 3. The results of active substances concentrations in different groups are shown in Table S1. All inoculation treatments affected the concentration of active substances in roots, with some groups showing an increase in the concentration of phenolic acids and tanshinones. In contrast, others showed a decrease (Fig. 7a and b). Overall, the total phenolic acid (TP, TP = SA + SB) concentrations in the plant roots showed a decreasing trend with increasing AMF species richness (Fig. 5e). In contrast, the total tanshinone (TTS, TTS = DH-I + TS-I + TS-IIA + CT) concentrations showed an increasing trend (Fig. 5f). The TP concentration in the roots was significantly higher (*p* < 0.05) than that in the CK group when the inoculum contained only one or two AMF(s). At the same time, it was greatly reduced when three or more AMFs were involved. In contrast, the concentration of TTS increased only when inoculated with three or four AMFs and decreased in other species richness (Fig. 7b). The results indicated that the concentration of *S. miltiorrhiza* root components was influenced by the AMF richness, but unlike DW, too many or too few AMFs were not conducive to TP or TTS concentrations.

Specifically, all mono-inoculation treatments effectively increased the TP concentration and reached a significant level (*p* < 0.05) (except for strain B). TP concentration increased when the two AMFs were co-inoculated, except for communities CE and DE. Unlike the biomass change, the concentration of TP increased after any combination of strains A, B, and C (from the same family), but the growth rate was lower than that of strain A or C alone. Similarly, the effect of inoculations with two AMFs was not higher than that of mono-inoculum in increasing TP concentration. For example, plant inoculation with strain A had the most potent effect on increasing the TP concentration, but the effect was reduced when combined with other AMFs. Also, after co-inoculation of strain E with C or D, the TP concentration was even lower than that of the CK. The combination of three AMFs from the same family (community ABC) had little effect on TP promotion; the combination of the close or distant family primarily increased TP concentration (e.g., communities ABD and BCE), but the effect was limited; on the contrary, TP concentration decreased when inoculated with the combination of three families (e.g., communities ADE, BDE, and CDE). When co-inoculated with more than three AMFs, community ABCE had the most potent enhancement effect, while communities ABCD and ABCDE reduced the concentration of TP by more than 12%. Interestingly, in contrast to root FW and DW, TP concentration increased when community ACDE was inoculated.

Contrary to the concentration of TP, most groups seem to be not conducive to the increase of tanshinone constituent concentrations (Table S1). The concentration of TTS was lower than that of CK by more than 36.3% (except for strain B) after inoculating the four AMFs alone. The total tanshinone concentration was lower after the combining of any two AMFs (more than 42.6%). When inocula were composed of three AMFs, TTS concentration increased in the same family (community ABC), mainly decreased in the close family combination (e.g., communities ABE and ACE), increased in the distant family combination (e.g., communities ABD, ACD, and BCD), and decreased in all three family combinations communities (e.g., ADE, BDE, and CDE). When there were more than three AMFs in the community, communities ABDE and ACDE increased the TTS concentration, with the increase of community ACDE reaching 77.4% (*p* < 0.05). In contrast, the TTS concentration decreased in other communities.

In summary, strain B increased the concentration of TP and TTS simultaneously; the combination of two AMFs decreased the concentration of TTS but increased the concentration of TP in most groups. When the three AMFs were combined, the two families with distant relationships seemed to be more conducive to increasing TP and TTS concentrations. When there were more than three AMF species in communities, only the communities ABDE and ACDE increased the concentration of TP and TTS simultaneously. FW and DW, TP, and TTS concentrations were influenced by AMF species richness and member affinities in the community. The co-inoculation of three to four more distant AMFs showed a greater potential in enhancing the concentration of active ingredients.

### The accumulation of active substances

**Fig. 8:**
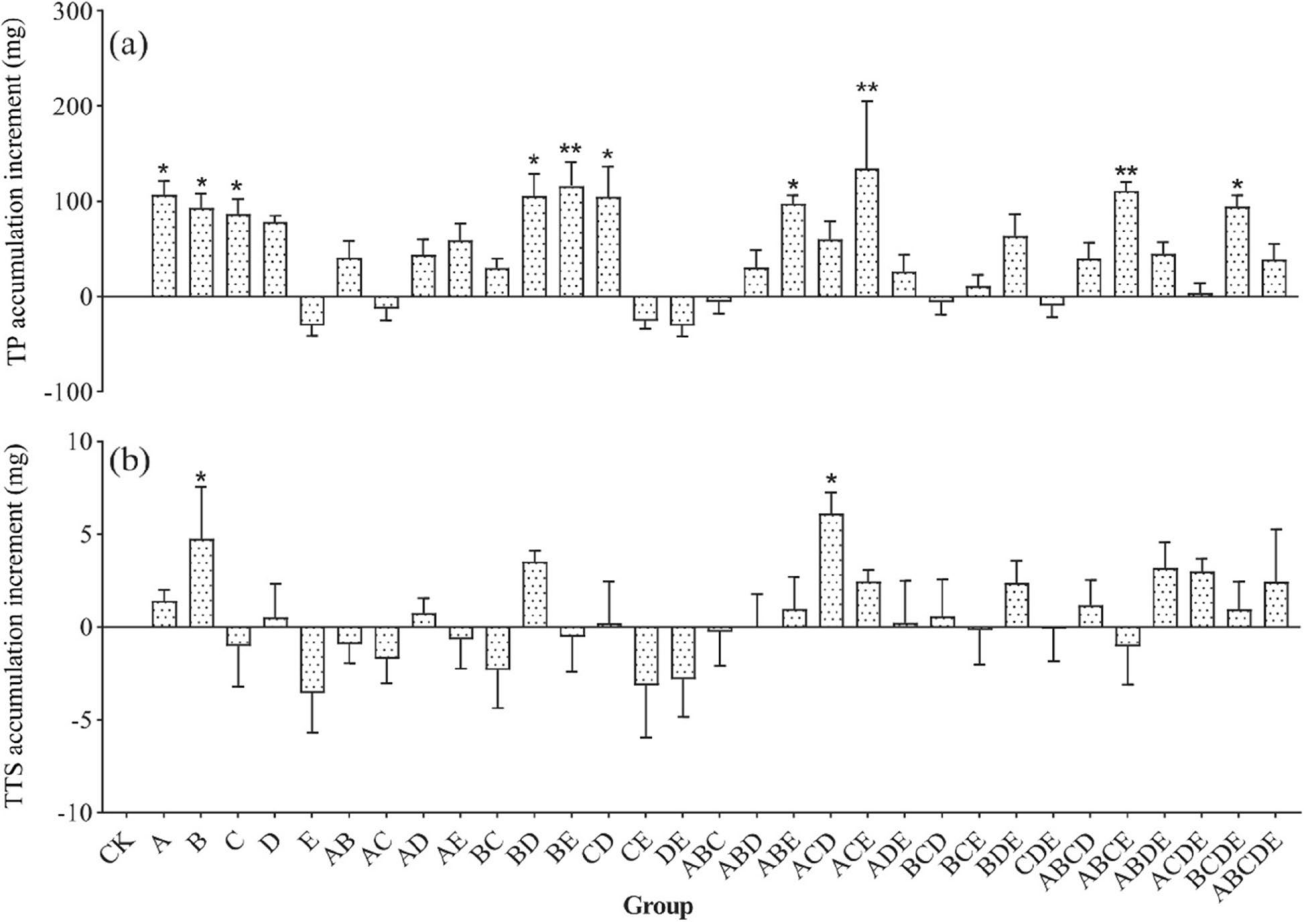
The increase of TP and TTS accumulations in *S. miltiorrhiza* root compared with CK. (**a**) the TP accumulation and (**b**) the TTS accumulation. Different compositions of thirty-two groups were used in this study as Fig. 2b. "*" indicates a significant difference (*p* < 0.05), and "**" indicates a highly significant difference (*p* < 0.01) when compared with CK

The accumulation of bioactive constituents (concentration × DW, mg/plant) involves both biomass and concentration, which is the focus of the herbal medicine cultivation industry. To develop the potential of AMFs, the effects of five AMFs and twenty-six combinations on the accumulation of active ingredients were evaluated to identify inocula that enhance the potential economic yield of *S. miltiorrhiza* roots (Table S2). Different AMF inocula have different effects on the active ingredients. The accumulation of TP (ATP) and the accumulation of TTS (ATTS) increased in most groups (Fig. 8a and b). Inoculation treatments increased the levels of ATP and ATTS (Fig. 5g and h). When inoculum species contained one or four AMF(s), ATP increased significantly (*p* < 0.05), while ATTS increased with increasing AMF species richness. This suggests that the levels of ATP and ATTS were influenced by AMF species richness.

Mono-inoculation with strain B significantly increased ATP and ATTS (*p* < 0.05) by 91.7% and 85.1%, respectively. Inoculation with strains A or D also favored ATP and ATTS, but inoculation with strain E reduced the accumulation by more than 30.2%. In the mixed inoculation treatment, both communities BE and ACD simultaneously increased ATP and ATTS by more than 50% (Fig. 8). Notably, strain A and communities BE, ACE, and ABCE significantly increased ATP by 100%. Community ACD significantly increased ATTS by more than one time (*p* < 0.05). In addition to the effects on ATP and ATTS, most inoculation treatments also benefited from the accumulation of phenolic acids and tanshinone components (Table S2). For example, the community BE significantly increased the accumulation of DS by 236% (*p* < 0.05); community ACD significantly increased the accumulation of DH-I, TS-I, TS-IIA, and CT by more than 100%; community ACE significantly increased the accumulation of CA, RA, SB, SA, DT-I, and TS-I by more than 100% (*p* < 0.05). In addition, strains A and B and communities AD, BD, ABE, ACD, ACE, BDE, and BCDE simultaneously increased the accumulation of nine active ingredients, which has great potential to increase the economic yield of *S. miltiorrhiza* when inoculated with AMFs.

### Global statistical analyses

**Fig. 9:**
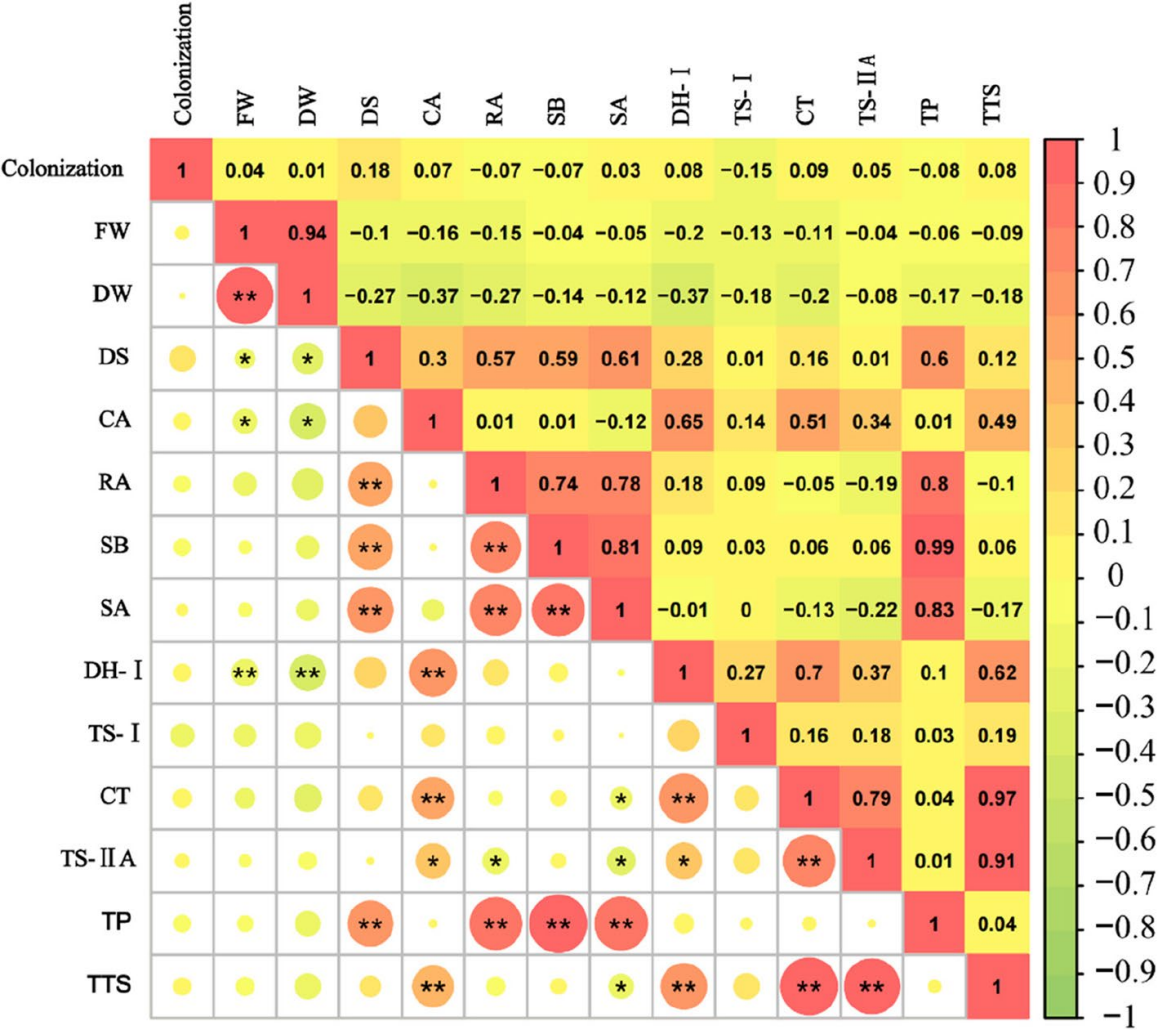
Correlation analysis of root indexes of *S. miltiorrhiza*. "*" indicates that there is a significant difference between the two indicators (*p* < 0.05), and "**" that there is a highly significant difference between the two indicators (*p* < 0.01)

The total correlations between the analyzed variables are shown in Fig. 9. The mycorrhizal colonization rate was positively correlated with FW, DW, and the concentrations of DS, CA, SA, DH-I, CT, TS-IIA, and TTS, while negatively correlated with RA, SB, TS-I and TP concentrations. The correlation coefficient was relatively small (*r* < 0.2), suggesting that there might not be a direct correlation between mycorrhizal colonization rate, biomass, and active component concentrations. Interestingly, both FW and DW were negatively correlated with the concentrations of nine components. Specifically, the FW and DW were significantly negatively correlated with DS and CA (*p* < 0.05) and highly significantly negatively correlated with DH-I (*p* < 0.01), which suggests that when there was a symbiotic relationship between AMF and plants, plants might reduce the accumulation of some dry matter to increase the synthesis of secondary metabolites without affecting their growth.

## Discussion

### Response of *S. miltiorrhiza* productivity to AMF inoculum

AMF colonization of roots was one of the oldest and most common interactions in ecology [33], facilitating plant uptake of macronutrients, especially in N and P [34–36], through the arbuscular (a specialized structure in the host root that was the site of nutrient exchange between the plant and the fungi). In return, plants allocate up to 20% of photosynthetic products (carbon sources) to AMF, thus ensuring their growth [37]. Root colonization occurs as a prerequisite for plant–microbe interactions. In our study, all inoculation treatments formed a good symbiosis with AMF(s). Still, there was not necessarily a correlation between the root colonization rate and FW or DW (Fig. 9). For example, when strain C was colonized in the root alone, the colonization rate was the lowest (54.83%). At the same time, the FW and DW still increased compared with CK. On the contrary, some groups with higher colonization rates (such as communities AC and CE) showed lower FW and DW. This has been shown in many studies [38, 39].

Due to the strong host specificity of AMF, it is necessary to explore the interaction characteristics between host plants and AMF(s). It has been widely reported that AMF forms symbiotic relationships with host plants and increases plant productivity [10, 40, 41], which is often attributed to AMF improving mineral nutrient uptake [42]. While some AMF communities in this study still had adverse effects on herb productivity, such as the inoculation of strain E and communities AC and DE. It seemed that the symbiotic benefits did not always outweigh the costs of AMF-plant symbiosis, meaning that not all AMF symbiosis was beneficial to plants but instead detrimental to plant productivity [43]. Nevertheless, most treatments still effectively promoted *S. miltiorrhiza* productivity, with DW significantly increased by more than 80% (*p* < 0.05) by inoculating communities BD, ABE, ACE, ABCE, and BCDE, which had great potential in the application of AMF(s) to the improvement of root yield. Interestingly, the combination of closer AMFs, such as any combination of inoculation with A, B, and C (from the same family), resulted in lower biomass than the mono-inoculation treatment. This was in agreement with Maherali et al. [44], indicating that when multiple AMFs of the same family are included in the inoculum, the growth-promoting effect on the plant is diminished due to competition between ecological niches caused by their functional redundancy [45]. We also found that overdispersal affinities in mixed communities (e.g., community ACDE) were also detrimental to root biomass, suggesting that AMF affinities influence plant growth in the community and that species compositions that are too close or distant are not conducive to productivity. Plant productivity also appears to be influenced by species richness [23], which increases with AMF species richness and has been confirmed in *Plantago lanceolata* [44]. This suggests that the productivity of *S. miltiorrhiza* is also influenced by both AMF species richness and affinities in the communities and that community members who are too close or too distant are not conducive to plant productivity. The growth promotion of AMF-plant symbiosis may be related to the fact that colonization significantly improved the growth status of plants, increased the net photosynthetic rate and antioxidant enzyme activity, and promoted the accumulation of soluble sugar, soluble protein, and proline in plants [46].

MD was often used to reflect the degree of compatibility between plants and AMF(s) and the effectiveness of mycorrhizal inoculum. In this study, the MD of most groups was more than 100%, which promoted root biomass (Table 2), and both communities ABE and ACE reached more than 200% of MDs, with significant plant growth promoting effect (*p* < 0.05). This further indicated that S*. miltiorrhiza* was a mycorrhizal-dependent medicinal plant [20], which could form a good symbiosis with different AMFs. Because of these effects on plant health and adaptation, AMF inoculum could be an essential strategy for sustainable agriculture [47].

### Response of bioactive constituent’s concentration to diversity

Secondary metabolites were the critical components of the interaction between plants and the environment to adapt to stresses and also the vital basis for the medicinal efficacy of herbal medicines. It is generally accepted that the chemical compositions of plants are directly or indirectly related to endophytic microbes and their interactions with host plants [48]. As the most reliable soil microbiome, AMF was intimately involved in plant physiological and biochemical metabolic processes promoting the production and accumulation of active components in medicinal plants, such as terpenoids, phenols, and alkaloids [49]. There is evidence that AMF colonization significantly alters the content or composition of secondary metabolites of aromatic plants [50, 51], which has been demonstrated in *Salvia officinalis* L. [52] and *Angelica archagelica* L. [53]. Mono-inoculation of *G. versiforme* or *Ac. Bireticulata* effectively improved the growth and increased the concentration and accumulation of tanshinones and SB in roots [18, 54]. This study showed that mono-inoculation of *G. formosanum*, *S. constrictum*, *R. manihotis*, *Ac. laevis* and *Am. Gerdemanni* significantly increased the concentration or accumulation of TP in roots (*p* < 0.05), but inoculation of *S. Constrictum* alone significantly promoted the level of ATTS. This study also showed that the concentration of TTS and TP in roots was influenced by AMF species richness, with an increase in species richness facilitating an increase in TTS concentration and a less favorable increase in the concentration of TP (Fig. 5e and f). Overall, simultaneous inoculation with three or four species was more advantageous for TP and TTS concentration. In addition, the concentrations of active ingredients were influenced by the affinities of AMFs in the community, and the combinations of species with more distant affinities (e.g., communities ABD and BCD) seemed to be more favorable for the enhancement of TP and TTS concentrations. Notably, both the more dispersed community ACDE and the closer community ABC increased both TP and TTS concentration and showed inhibitory effects on root biomass, suggesting that the symbiotic relationship between AMF communities and plants influences the plants to continuously balance growth and defense functions during growth and development, ultimately affecting the yield and quality of the medicinal plant.

Secondary metabolites are important phytochemical defense compounds produced during the interaction between plant-microorganism interactions [55]. Phenolic compounds, with high antioxidant potential [56], have several plant protection functions, such as resistance to pathogens invasion, osmotic regulation, and hormone production [57, 58]. Terpenoids are also often considered defense products of plants against fungal colonization [59]. AMF symbiosis has been shown to upregulate the transcription of genes encoding enzymes involved in isoprene-like biosynthesis and was strongly correlated with terpenoid concentrations [60]. However, the effect of AMF colonization on plant active ingredients varies depending on the plant as well as the fungal species [12, 54]; thus, the present study systematically investigated the inoculation effect of single to multiple AMFs on the active ingredients of *S. miltiorrhiza* and found that inoculation with strain A was most favorable for the concentration of TP, while community ACDE was most favorable for the concentration of TTS. The inoculation effect of AMF communities was influenced by the species richness and the members' affinity, and the simultaneous inoculation of three or four AMFs from different families with distant affinities was more favorable for the formation of TP and TTS concentrations. The mechanism of increased synthesis of phenolic acids and tanshinones after AMF colonization has been extensively studied. The increase in the concentration of phenolic acids can be attributed to the fact that AMF colonization activated the plant defense mechanism and induced the production of some signaling molecules, thus enhancing the activity of L-phenylalanine ammonia lyase and chalcone synthesis to promote the synthesis of phenolic compounds [61]. On the one hand, the increase in terpenoids may be due to the increased expression of genes encoding enzymes by AMFs, such as genes of the terpene synthase family, 1-deoxy-d-xylalose5-phospho synthase, and 1-deoxy-d-xylalose-5-phosphate reductoisomerase [62, 63]. On the other hand, it can alter the levels of plant hormones, such as jasmonic acid, gibberellic acid, gibberellic acid, and 6-benzylaminopurine, and lead to transcriptional activation of sesquiterpene biosynthesis gene expression [13]. Our results show that the inoculation of a suitable AMF community has great potential to improve the bioactive components of *S. miltiorrhiza*.

### Difference in response to plant growth and secondary metabolism between a single AMF and AMF communities

Several recent studies have suggested that microbial communities provide additional ecosystem functions and services, including plant-promoting and plant-protective effects. For example, when functionally complementary AMF species colonized plant roots, the colonization rate of mixed inocula was higher than that of a mono-inoculum [21, 64, 65]. Conversely, certain single species have been shown to be more favorable for plant growth [66], and increasing community diversity did not result in greater benefits [67, 68]. Research on the role of microbes in promoting plant growth has gradually shifted from mono-inoculum to microbial communities in recent years [69–71]. Despite the widely established beneficial effects of AMF on plant growth and metabolism, the inoculation effects of a single AMF or communities were inconsistent and highly variable on plants. Therefore, this study systematically explored the responses of mono-inoculum to mixed inocula and found that all inoculation groups formed a good symbiosis with *S. miltiorrhiza* (up to 89.97% colonization rate), and the mixed inoculation treatments possessed higher mycorrhizal colonization rates compared to mono-inoculum treatments (Fig. 5a) [72]. Differences in colonization rate between communities may be related to multiple group interactions [73].

Consistent with most studies, our results also indicate that mixed inocula were more beneficial to plant growth than mono-inoculum (Fig. 5b and c) [72, 74], but most current studies were limited to two or three AMFs [75, 76], ignoring the fact that AMFs were complex and diverse in the field environment. In this study, communities BD, BE, ABE, ACE, ABCE, BCDE, and ABCDE had obvious enhancement benefits for both DW and FW than a single inoculum. This beneficial effect may result from functional diversity among the species, allowing more efficient use of resources through their different ecological niches [22]. AMF formed an extensive mycelial network underground, which greatly enhanced the uptake of water and minerals (such as phosphorus, nitrogen, potassium, calcium, sulfur, zinc, and copper) by roots, altered the soil structure, improved soil fertility and promoted the plant growth [77, 78]. In contrast, the remaining AMF inocula mixtures did not show synergistic effects on plant growth, which may be attributed to the fact that one of the species may have become dominant [68], or it may be related to complex interactions between species or competition for similar ecological niches [79].

In addition to differences in the regulation of plant growth, secondary metabolites also varied depending on the inoculum. For example, the combined inoculation of *Cynara cardunculus* L. var. scolymus with *G. intraradices* and *G. mosseae* under field conditions was more beneficial in increasing total phenolic acid concentration in leaves and flowers than mono-inoculation [80]. The AMF communities in this study did not appear to be more effective than mono-inoculum in increasing the TP concentration (Fig. 5e), while the AMF communities seemed to have more potential in increasing TTS concentration (Fig. 5f). Strain B and communities ABC, ABD, BCD, BCE, ABDE, and ACDE increased TP and TTS concentrations. Communities ABD, BCE, and ABDE increased plant FW and DW, while some communities did not show simultaneous promoting effects, which could be attributed to the antagonistic solid effect between species, suggesting that the effectiveness of mixed inocula might be related to the compatibility between different microorganisms [65]. These variations further indicate that the mycorrhizal benefits largely depend on plant-fungi combinations [81]. Although only a few communities were able to increase the yield of medicinal parts of *S. miltiorrhiza* and the concentration of secondary metabolites more effectively than a single AMF, we still believed that higher AMF richness might have more potential in promoting *S. miltiorrhiza* growth and yield, especially when three to four distantly related AMF species were co-inoculated. This might be attributed to the fact that biodiversity is essential for maintaining ecosystem function, and communities with higher richness might contain more ecological functions with specific buffering properties, which could maintain ecosystem stability and function in more complex environments [82, 83].

### The key to AMF as a biofertilizer

Biofertilizers could improve soil fertility and achieve higher yields and quality without adversely affecting the agricultural environment, so they have excellent prospects in herbal cultivation [84, 85]. Despite the widely recognized efficacy of microbial fertilizers, it was a fact that biofertilizers sometimes provided little benefit in promoting growth [43, 86]. Several pieces of evidence show that the introduction of foreign AMFs does not bring more benefits than native AMFs [87, 88], because they have to compete with native AMFs [89]. This may explain why most commercial inocula have little effect on plant growth promotion. More interestingly, some researchers have found significant differences in the effects of using the same inoculum in different locations [90, 91], making AMFs much less effective as biofertilizers. Therefore, we designed experiments in this study using native AMFs to maximize biofertilizers' effectiveness. In this study, stain B, communities BD, ACD, ACE, and BDE effectively promoted biomass (more than 60%) and increased the ATP and ATTS as well as the accumulation of active ingredients, which can be considered as potential communities for AMF application to improve the economic yield of *S. miltiorrhiza*, with communities ACD and ACE having more potential.

Biodiversity affects many ecosystem functions, and plant productivity often increases with increasing species diversity [92]. This positive effect could often be explained by functional diversity, where different species occupy different ecological niches and perform different functions [93]. The results of this study showed an increasing trend of FW, DW and ATTS with increasing species richness (Fig. 5b, c, and h), while ATP showed fluctuations, with greater benefits when a single or four AMF species were co-inoculated (Fig. 5g). All these results strongly emphasize that the AMF species richness influenced the inoculation benefits of AMFs in the inoculum and that mixed AMF inocula showed greater application potential than mono-inoculum. In addition, the plant growth promotion effect, as well as lower ATP and ATTS of any inocula of strains A, B, and C (with the same family), was lower than that of mono-inoculum (Figs. 6 and 8), indicating that closer species may have similar ecosystem functions and competitive effects and that increasing the number of functionally redundant AMF in the community will not bring more benefits to plants [77]. There were positive or negative effects of the different family combination communities on root growth, depending on the species composition, which may be related to the complex interactions between different species [79]. However, in general, the more distantly related combinations of two-family AMFs (e.g., communities ACD and ACE) better promoted *S. miltiorrhiza* root growth and facilitated TP and TTS accumulation (Figs. 6 and 8), showing a better potential for application.

Overall, the application of AMF did not always promote the growth and bioactive production of *S. miltiorrhiza*, while the inoculum benefit of mixed inocula appeared to be higher than that of single inoculum. The inoculum benefits of mixed inocula were limited by the AMF species richness and member affinities in the community. Therefore, we believe that the key to biofertilization is to select candidates from native AMFs and to select three to four distantly related AMFs in combination according to their phylogenetic relationships as microbial inocula to ensure the yield of *S. miltiorrhiza.* In addition, as the most crucial microbiome of biofertilizers, AMF plays a vital role in improving and maintaining soil structure, plant community structure and diversity, promoting nutrient cycling and transformation, and reducing nutrient loss, thus contributing to the long-term sustainability of the ecosystem [94–96]. It can be seen that the use of AMF as a biofertilizer is an eco-technical tool for achieving agricultural sustainability.

### Conclusion

In this study, we systematically explored the impacts of inoculating one to five AMFs on the yield of medicinal parts and the accumulation of secondary metabolites of *S. miltiorrhiza*. It was found that the growth-promoting effect of AMF communities was more potent than mono-inoculum in promoting the growth and secondary metabolite accumulation of *S. miltiorrhiza* roots. In addition, there was a correlation between plant productivity and economic yield with the richness and affinities of AMF species in the community. Three or four distant AMFs were co-inoculated to enhance plant productivity and economic yield, among which the communities ACD and ACE showed a high potential for practical application. This suggests that the colonization of AMF communities is an important strategy to improve the ecological cultivation of herbs, but more practical AMF biofertilizers should be developed, taking into account both the richness of AMFs in the community and the affinities of the constituent members, which is vital for the development of more sustainable agriculture and more efficient production of medicinal plant materials.

### Supplementary Information

**Additional file 1: Fig. S1.** Effect of AMFs inoculation on roots growth of *S. miltiorrhiza* seedlings after twenty-four weeks transplanting. **Table S1.** Effect of AMF inoculations on active substances concentrations (mg·g^-1^ DW). **Table S2.** Yield of active substances in *Salvia miltiorrhiza* root (mg DW).

## Data Availability

All data generated or analyzed during this study are included in this published article.

## Abbreviations

AMF: Arbuscular mycorrhizal fungi
SB: Salvianolic acid B
TS-IIA: Tanshinone IIA
DT-I: Dihydrotanshinone I
DS: Danshensu sodium
CA: Caffeic acid
RA: Rosmarinic acid
SA: Salvianolic acid A
CT: Cryptotanshinone
TS-I: Tanshinone I
FW: Fresh weight
DW: Dry weight
TP: Total phenolic acid
TTS: Total tanshinone
ATP: Total phenolic acid accumulations
ATTS: Total tanshinone accumulations
