# Supplementary material for: Effect the accumulation of bioactive constituents of a medicinal plant (Salvia Miltiorrhiza Bge.) by arbuscular mycorrhizal fungi community
Source: BMC Plant Biol. 2023 Nov 28;23:597. doi: 10.1186/s12870-023-04608-x (PMC10683245; doi:10.1186/s12870-023-04608-x)
Supplement: Supplementary file 1 — Additional file 1: Fig. S1. Effect of AMFs inoculation on roots growth of S. miltiorrhiza seedlings after twenty-four weeks transplanting. Table S1. Effect of AMF inoculations on active substances concentrations (mg·g-1 DW). Table S2. Yield of active substances in Salvia miltiorrhiza root (mg DW). [file 12870_2023_4608_MOESM1_ESM.docx]

**
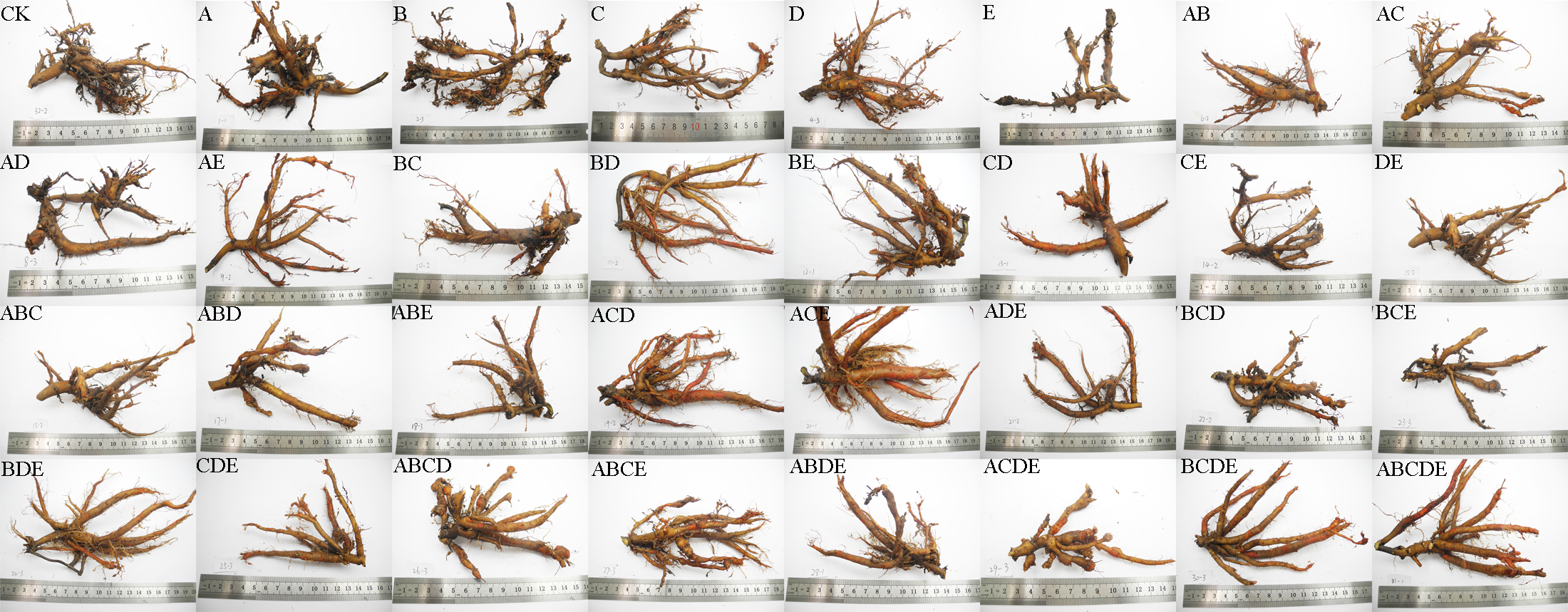
**

**Fig. S1** Effect of AMFs inoculation on roots growth of *S. miltiorrhiza* seedlings after twenty-four weeks transplanting.

**Table S1** Effect of AMF inoculations on active substances concentrations (mg·g^-1^ DW).

| Number | Group | DSS | CA | RA | SB | SA | DH Ⅰ | TS Ⅰ | CT | TSⅡA | TP | TTS |
| --- | --- | --- | --- | --- | --- | --- | --- | --- | --- | --- | --- | --- |
| S1 | A | 0.14±0.03 | 0.06±0.03 | 4.15±0.50 | 45.01±3.90** | 0.44±0.03** | 0.05±0.02 | 0.009±0.003 | 0.88±0.62 | 0.59±0.33 | 45.45±3.913** | 1.52±0.98 |
| S2 | B | 0.13±0.03 | 0.04±0.00 | 3.22±1.02 | 36.40±4.81 | 0.33±0.04 | 0.04±0.02 | 0.007±0.007 | 1.04±1.06 | 0.59±0.48 | 36.73±4.84 | 1.67±1.55 |
| S3 | C | 0.08±0.02* | 0.04±0.00 | 3.31±0.68 | 38.90±5.86** | 0.33±0.07 | 0.03±0.01 | 0.008±0.001 | 0.52±0.12 | 0.41±0.09 | 39.23±5.92** | 0.97±0.20 |
| S4 | D | 0.11±0.05 | 0.04±0.01 | 2.44±0.55 | 36.64±2.01* | 0.30±0.05 | 0.03±0.00 | 0.007±0.003 | 0.77±0.24 | 0.53±0.18 | 36.94±2.03* | 1.33±0.41 |
| S5 | E | 0.15±0.05 | 0.07±0.02* | 4.76±0.43** | 41.23±8.27* | 0.45±0.12** | 0.06±0.01 | 0.013±0.002** | 0.71±0.14 | 0.43±0.13 | 41.67±8.39** | 1.21±0.28 |
| S6 | AB | 0.11±0.02 | 0.04±0.01 | 3.63±0.63 | 36.61±4.55* | 0.39±0.08* | 0.04±0.02 | 0.008±0.002 | 0.71±0.30 | 0.43±0.17 | 37.01±4.63* | 1.18±0.47 |
| S7 | AC | 0.12±0.04 | 0.05±0.02 | 3.55±1.64 | 34.84±7.91 | 0.39±0.11* | 0.06±0.03 | 0.008±0.003 | 0.94±0.61 | 0.51±0.48 | 35.23±8.01 | 1.52±1.06 |
| S8 | AD | 0.11±0.04 | 0.04±0.01 | 2.51±0.72 | 30.82±3.51 | 0.33±0.08 | 0.04±0.01 | 0.009±0.002 | 0.78±0.36 | 0.53±0.28 | 31.16±3.58 | 1.35±0.64 |
| S9 | AE | 0.16±0.02 | 0.04±0.01 | 3.87±1.03 | 41.98±5.64** | 0.44±0.08** | 0.03±0.01 | 0.007±0.003 | 0.72±0.09 | 0.55±0.26 | 42.42±5.72** | 1.31±0.34 |
| S10 | BC | 0.11±0.02 | 0.03±0.01 | 2.86±0.24 | 35.10±2.04 | 0.36±0.03 | 0.02±0.00 | 0.008±0.003 | 0.45±0.14* | 0.40±0.08 | 35.46±2.05 | 0.88±0.22 |
| S11 | BD | 0.09±0.06 | 0.04±0.01 | 2.84±0.84 | 33.59±3.02 | 0.30±0.08 | 0.03±0.01 | 0.005±0.003 | 0.82±0.36 | 0.57±0.21 | 33.89±3.04 | 1.42±0.58 |
| S12 | BE | 0.26±0.11** | 0.04±0.01 | 3.94±1.33 | 39.89±8.31** | 0.49±0.13** | 0.03±0.00 | 0.009±0.001 | 0.53±0.14 | 0.40±0.15 | 40.38±8.44** | 0.96±0.29 |
| S13 | CD | 0.12±0.03 | 0.03±0.01 | 3.15±0.77 | 37.16±2.35* | 0.41±0.01* | 0.03±0.01 | 0.006±0.002 | 0.65±0.22 | 0.47±0.16 | 37.57±2.35* | 1.16±0.33 |
| S14 | CE | 0.11±0.02 | 0.06±0.03* | 2.72±0.83 | 26.26±1.90 | 0.26±0.02 | 0.06±0.03 | 0.006±0.004 | 0.69±0.45 | 0.36±0.23 | 26.51±1.91 | 1.11±0.72 |
| S15 | DE | 0.12±0.02 | 0.07±0.03** | 3.24±0.63 | 29.77±3.78 | 0.35±0.08 | 0.07±0.01 | 0.009±0.003 | 0.74±0.14 | 0.37±0.09 | 30.13±3.85 | 1.19±0.15 |
| S16 | ABC | 0.09±0.01 | 0.07±0.02** | 3.44±0.73 | 31.08±3.81 | 0.31±0.03 | 0.11±0.07** | 0.010±0.003* | 1.18±0.62 | 0.49±0.17 | 31.39±3.82 | 1.79±0.82 |
| S17 | ABD | 0.08±0.01* | 0.06±0.02 | 2.94±0.48 | 34.84±2.98 | 0.36±0.03 | 0.04±0.02 | 0.007±0.001 | 0.87±0.27 | 0.64±0.11 | 35.2±3.01 | 1.57±0.41 |
| S18 | ABE | 0.12±0.07 | 0.06±0.02 | 2.41±0.79* | 31.49±7.11 | 0.30±0.08 | 0.04±0.02 | 0.007±0.001 | 0.67±0.27 | 0.39±0.21 | 31.79±7.19 | 1.10±0.42 |
| S19 | ACD | 0.10±0.03 | 0.05±0.01 | 1.97±0.76** | 30.03±4.43 | 0.24±0.07 | 0.07±0.04 | 0.009±0.002 | 1.22±0.76 | 0.80±0.38* | 30.27±4.49 | 2.09±1.17 |
| S20 | ACE | 0.10±0.03 | 0.04±0.02 | 2.51±1.27 | 30.54±8.48 | 0.31±0.11 | 0.04±0.02 | 0.007±0.003 | 0.68±0.14 | 0.36±0.10 | 30.84±8.59 | 1.08±0.07 |
| S21 | ADE | 0.10±0.02 | 0.08±0.05* | 2.23±0.54* | 29.36±3.82 | 0.26±0.08 | 0.05±0.03 | 0.006±0.001 | 0.79±0.33 | 0.55±0.24 | 29.62±3.79 | 1.39±0.60 |
| S22 | BCD | 0.14±0.02 | 0.09±0.03** | 3.13±1.15 | 34.91±5.20 | 0.32±0.03 | 0.07±0.02 | 0.007±0.002 | 1.39±0.07 | 0.71±0.06 | 35.23±5.22 | 2.18±0.14 |
| S23 | BCE | 0.15±0.02 | 0.08±0.02** | 2.13±0.44** | 33.22±4.40 | 0.33±0.04 | 0.06±0.03 | 0.008±0.001 | 1.01±0.19 | 0.56±0.05 | 33.55±4.42 | 1.64±0.22 |
| S24 | BDE | 0.08±0.03* | 0.05±0.01 | 1.94±0.65** | 29.43±5.24 | 0.29±0.11 | 0.05±0.03 | 0.008±0.001 | 0.83±0.32 | 0.55±0.15 | 29.71±5.34 | 1.43±0.50 |
| S25 | CDE | 0.06±0.01** | 0.05±0.01 | 1.49±0.31** | 21.46±3.64** | 0.2±0.070* | 0.04±0.01 | 0.008±0.001 | 0.79±0.28 | 0.52±0.12 | 21.66±3.7** | 1.36±0.39 |
| S26 | ABCD | 0.07±0.01* | 0.05±0.01 | 2.03±0.41** | 26.82±2.66 | 0.24±0.04 | 0.04±0.01 | 0.008±0.001 | 0.73±0.10 | 0.55±0.07 | 27.06±2.65 | 1.33±0.17 |
| S27 | ABCE | 0.09±0.02 | 0.03±0.00 | 2.87±0.25 | 35.03±5.18 | 0.36±0.04 | 0.03±0.01 | 0.008±0.002 | 0.43±0.02* | 0.28±0.06 | 35.39±5.22 | 0.75±0.07* |
| S28 | ABDE | 0.07±0.05* | 0.06±0.02 | 2.35±0.96* | 30.97±6.15 | 0.27±0.08 | 0.06±0.03 | 0.006±0.002 | 1.12±0.47 | 0.73±0.25 | 31.24±6.23 | 1.92±0.74 |
| S29 | ACDE | 0.15±0.04 | 0.08±0.02** | 2.60±0.54 | 32.86±4.75 | 0.31±0.05 | 0.10±0.04** | 0.007±0.004 | 1.72±0.63** | 0.88±0.25** | 33.17±4.8 | 2.71±0.91** |
| S30 | BCDE | 0.08±0.03* | 0.03±0.01 | 2.67±0.73 | 31.69±2.36 | 0.32±0.06 | 0.04±0.01 | 0.008±0.003 | 0.61±0.15 | 0.38±0.05 | 32.02±2.42 | 1.04±0.20 |
| S31 | ABCDE | 0.08±0.04* | 0.05±0.01 | 1.66±0.90** | 24.54±7.74* | 0.26±0.13 | 0.05±0.01 | 0.008±0.001 | 0.75±0.18 | 0.43±0.15 | 24.8±7.87* | 1.24±0.33 |
| S32 | NM | 0.12±0.02 | 0.04±0.02 | 3.34±0.74 | 30.75±2.99 | 0.31±0.03 | 0.05±0.02 | 0.007±0.003 | 0.94±0.59 | 0.53±0.47 | 31.06±3.02 | 1.53±1.04 |

**Table S2** Yield of active substances in *Salvia miltiorrhiza* root (mg·DW).

| Number | Group | DSS | CA | RA | SB | SA | DH Ⅰ | TS Ⅰ | CT | TSⅡA | TP | TTS |
| --- | --- | --- | --- | --- | --- | --- | --- | --- | --- | --- | --- | --- |
| S1 | A | 0.64±0.25 | 0.26±0.15* | 19.20±6.51* | 206.11±58.48* | 2.00±0.59* | 0.21±0.12 | 0.039±0.016 | 4.02±3.07 | 2.71±1.67 | 208.11±59.04* | 6.99±4.87 |
| S2 | B | 0.76±0.36* | 0.23±0.08 | 16.29±4.47 | 192.41±50.58* | 1.78±0.59 | 0.19±0.12 | 0.039±0.035 | 6.57±7.93* | 3.52±3.68 | 194.19±51.14* | 10.32±11.75* |
| S3 | C | 0.36±0.10 | 0.19±0.06 | 15.83±4.56 | 186.63±49.20* | 1.60±0.52 | 0.17±0.07 | 0.039±0.013 | 2.43±0.54 | 1.92±0.31 | 188.24±49.71* | 4.55±0.89 |
| S4 | D | 0.56±0.46 | 0.20±0.10 | 11.98±5.43 | 178.02±63.16 | 1.48±0.76 | 0.14±0.04 | 0.033±0.017 | 3.48±0.94 | 2.43±0.78 | 179.5±63.89 | 6.09±1.67 |
| S5 | E | 0.25±0.13 | 0.13±0.07 | 8.04±2.670 | 69.94±28.34 | 0.76±0.35 | 0.10±0.03 | 0.021±0.006 | 1.19±0.45 | 0.72±0.33 | 70.7±28.68 | 2.03±0.81 |
| S6 | AB | 0.41±0.22 | 0.14±0.05 | 13.82±8.19 | 140.28±80.58 | 1.53±0.97 | 0.15±0.04 | 0.028±0.006 | 2.77±2.20 | 1.69±1.38 | 141.81±81.55 | 4.63±3.59 |
| S7 | AC | 0.29±0.08 | 0.12±0.04 | 8.68±3.60 | 87.80±22.23 | 0.98±0.26 | 0.14±0.08 | 0.020±0.010 | 2.36±1.65 | 1.33±1.30 | 88.78±22.48 | 3.85±2.90 |
| S8 | AD | 0.50±0.18 | 0.16±0.08 | 11.56±4.30 | 144.03±57.52 | 1.54±0.57 | 0.17±0.08 | 0.046±0.027* | 3.63±2.53 | 2.48±1.89 | 145.57±58.07 | 6.33±4.52 |
| S9 | AE | 0.60±0.31 | 0.15±0.08 | 13.34±3.87 | 158.85±81.29 | 1.62±0.71 | 0.13±0.05 | 0.025±0.008 | 2.73±1.39 | 2.04±1.27 | 160.47±81.99 | 4.92±2.64 |
| S10 | BC | 0.44±0.22 | 0.12±0.04 | 10.61±3.68 | 130.28±47.64 | 1.35±0.59 | 0.09±0.04 | 0.030±0.015 | 1.68±0.76 | 1.46±0.45 | 131.64±48.23 | 3.26±1.26 |
| S11 | BD | 0.52±0.40 | 0.22±0.12 | 17.07±7.02 | 205.03±68.13* | 1.81±0.67 | 0.20±0.09 | 0.027±0.004 | 5.26±3.46 | 3.60±2.16 | 206.84±68.58* | 9.09±5.70 |
| S12 | BE | 1.31±0.46** | 0.23±0.10 | 20.22±6.06* | 215.70±72.44* | 2.6±0.84** | 0.15±0.07 | 0.049±0.021** | 2.83±0.93 | 2.03±0.68 | 218.3±73.26** | 5.06±1.66 |
| S13 | CD | 0.63±0.23 | 0.17±0.07 | 17.05±9.15 | 204.05±113.68* | 2.25±1.21** | 0.14±0.06 | 0.032±0.016 | 3.27±1.33 | 2.34±0.76 | 206.31±114.88* | 5.79±2.01 |
| S14 | CE | 0.32±0.21 | 0.15±0.08 | 7.97±5.30 | 75.07±48.20 | 0.74±0.50 | 0.14±0.11 | 0.013±0.010 | 1.50±1.01 | 0.77±0.43 | 75.81±48.69 | 2.42±1.56 |
| S15 | DE | 0.29±0.16 | 0.15±0.08 | 7.73±4.62 | 69.56±36.64 | 0.83±0.48 | 0.16±0.09 | 0.020±0.011 | 1.74±0.97 | 0.85±0.44 | 70.4±37.12 | 2.78±1.40 |
| S16 | ABC | 0.28±0.10 | 0.21±0.10 | 10.42±3.80 | 94.65±34.09 | 0.93±0.36 | 0.32±0.22* | 0.029±0.008 | 3.52±2.03 | 1.45±0.63 | 95.58±34.42 | 5.31±2.79 |
| S17 | ABD | 0.28±0.16 | 0.20±0.09 | 10.94±6.28 | 130.41±81.75 | 1.35±0.86 | 0.13±0.06 | 0.027±0.015 | 3.10±1.54 | 2.33±1.24 | 131.76±82.61 | 5.59±2.82 |
| S18 | ABE | 0.69±0.29* | 0.34±0.07** | 14.95±4.79 | 196.81±54.6* | 1.88±0.65 | 0.21±0.05 | 0.046±0.015** | 3.94±1.20 | 2.36±1.19 | 198.69±55.25* | 6.55±1.87 |
| S19 | ACD | 0.51±0.21 | 0.28±0.11** | 10.83±6.39 | 160.19±59.82 | 1.28±0.59 | 0.36±0.27** | 0.049±0.023** | 6.78±5.17* | 4.50±2.84** | 161.47±60.37 | 11.68±8.24* |
| S20 | ACE | 0.67±0.47 | 0.31±0.21** | 21.23±25.79** | 233.09±211.14* | 2.40±2.39** | 0.30±0.31* | 0.046±0.026* | 5.23±4.62 | 2.46±1.24 | 235.49±213.52** | 8.04±5.87 |
| S21 | ADE | 0.45±0.24 | 0.30±0.16** | 9.41±5.25 | 125.91±65.81 | 1.18±0.84 | 0.21±0.11 | 0.026±0.019 | 3.28±1.50 | 2.28±1.05 | 127.1±66.58 | 5.79±2.64 |
| S22 | BCD | 0.39±0.23 | 0.26±0.19* | 7.87±3.74 | 94.21±52.94 | 0.88±0.54 | 0.20±0.14 | 0.018±0.010 | 3.92±2.57 | 1.99±1.27 | 95.08±53.46 | 6.13±3.98 |
| S23 | BCE | 0.48±0.17 | 0.25±0.05* | 7.40±3.78 | 111.07±41.51 | 1.13±0.49 | 0.18±0.05 | 0.028±0.010 | 3.29±0.89 | 1.91±0.83 | 112.2±41.99 | 5.40±1.66 |
| S24 | BDE | 0.43±0.32 | 0.25±0.13* | 10.79±7.83 | 163.27±93.65 | 1.62±1.20 | 0.26±0.22 | 0.043±0.022* | 4.59±2.69 | 3.05±1.60 | 164.89±94.81 | 7.94±4.48 |
| S25 | CDE | 0.26±0.20 | 0.21±0.11 | 6.32±4.28 | 90.86±58.11 | 0.88±0.66 | 0.17±0.10 | 0.033±0.018 | 3.18±1.69 | 2.10±1.02 | 91.74±58.76 | 5.48±2.77 |
| S26 | ABCD | 0.35±0.17 | 0.25±0.13* | 10.71±6.24 | 139.84±72.59 | 1.26±0.67 | 0.22±0.13 | 0.041±0.021* | 3.69±1.89 | 2.79±1.36 | 141.09±73.2 | 6.74±3.37 |
| S27 | ABCE | 0.53±0.11 | 0.15±0.02 | 17.60±5.91 | 209.86±55.21** | 2.15±0.54* | 0.18±0.08 | 0.050±0.015** | 2.59±0.69 | 1.70±0.45 | 212.01±55.74** | 4.52±1.17 |
| S28 | ABDE | 0.34±0.20 | 0.27±0.11* | 10.75±3.42 | 144.78±27.05 | 1.24±0.35 | 0.26±0.14 | 0.030±0.014 | 5.13±1.74 | 3.34±0.92 | 146.02±27.32 | 8.76±2.74 |
| S29 | ACDE | 0.43±0.08 | 0.24±0.10* | 8.37±4.22 | 104.26±45.81 | 0.99±0.47 | 0.32±0.18* | 0.025±0.019 | 5.42±3.10 | 2.80±1.44 | 105.25±46.28 | 8.56±4.71 |
| S30 | BCDE | 0.48±0.25 | 0.17±0.12 | 16.01±5.35 | 193.47±61.75* | 1.99±0.81* | 0.23±0.12 | 0.049±0.020** | 3.88±1.96 | 2.36±0.94 | 195.46±62.54* | 6.52±3.01 |
| S31 | ABCDE | 0.42±0.16 | 0.32±0.19** | 8.54±2.97 | 138.78±47.97 | 1.37±0.49 | 0.26±0.10 | 0.049±0.028** | 4.82±3.15 | 2.88±2.21 | 140.16±48.38 | 8.01±5.47 |
| S32 | NM | 0.39±0.17 | 0.11±0.05 | 10.55±4.95 | 100.32±48.91 | 0.99±0.45 | 0.14±0.08 | 0.023±0.017 | 3.31±3.39 | 2.10±2.55 | 101.31±49.36 | 5.58±6.00 |
